# The effect of wildfire air pollution on local hospital admissions in New York

**DOI:** 10.1088/2515-7620/ae69a4

**Published:** 2026-05-15

**Authors:** Mahdieh Danesh Yazdi, Nozomi Sasaki, Minghao Qiu, Guanyu Huang, Perry E Sheffield

**Affiliations:** 1 Program in Public Health, Stony Brook University, Stony Brook, NY, United States of America; 2 Department of Family, Population, and Preventive Medicine, Stony Brook University, Stony Brook, NY, United States of America; 3 School of Marine and Atmospheric Sciences, Stony Brook University, Stony Brook, NY, United States of America; 4 Icahn School of Medicine at Mount Sinai, New York, NY, United States of America

**Keywords:** air pollution, wildfires, PM_2.5_, cardiovascular disease, respiratory disease

## Abstract

Exposure to wildfire-associated smoke has increased in recent years in the Northeast of the United States. Of particular note was the smoke event in June 2023 when plumes from Canadian wildfires caused a high pollution event in New York State. We used data from Stony Brook Hospital to assess the effect of the June 2023 smoke event on healthcare encounters, including emergency department (ED) visits and inpatient admissions. We examined all-cause encounters, cardiovascular diseases (CVDs), hypertension, respiratory diseases, and asthma. We then extended our study to look at longer term trends using a time-series analysis looking at the effect of exposure to wildfire smoke-associated PM_2.5_ on healthcare encounters. We studied the association between exposure to wildfire-associated PM_2.5_ at the county level to daily hospital visits between January 2014 through July 2023. Other exposures of interest included non-smoke PM_2.5_, ozone, and temperature. We found increased rates of total hospital visits for CVD (rate ratio: 1.34 (95% CI: 1.07–1.68)) and hypertension (rate ratio: 1.47, (95% CI: 1.08–2.00)) during the smoke event in 2023 as compared to the reference period, driven primarily by increased ED visits. We also found an increase in the rate of inpatient respiratory admissions (rate ratio: 1.60 (195% CI: 1.03–2.48) as compared to the reference period. Our time-series analysis showed an increased rate of total encounters with exposure to wildfire-smoke PM_2.5_. Higher temperatures were also associated with increased rates of all-cause health care encounters as well as cardiovascular and respiratory encounters. Our study found adverse outcomes related to exposure to wildfire-associated air pollution in a suburban community during a high exposure wildfire smoke event. It highlights the need for further local mitigation and adaptation measures in response to increasing wildfires.

## 1. Introduction

Wildfire-generated air pollution has been a serious threat to human health and ecosystems in the United States (US) for several decades. Wildfire-smoke exposure has been extensively linked to adverse health outcomes, including but not limited to increased mortality and increased rates of cardiovascular and respiratory disease [1–10]. With the effects of climate change expected to exacerbate wildfires, a better understanding of the specific health effects of wildfires and the variation of those effects by region and population will inform mitigation and adaptation measures. Climate change is expected to increase the length and severity of droughts, leading to prolonged wildfire seasons and more intense wildfires [11–14]. These effects have been predominantly experienced and studied in the Western US and are increasingly being seen in other regions as well [15, 16].

More recently, the Northeast region of the US has been affected by wildfires in significant ways. There has been an increase in wildfire-generated air pollution that begins in Canada and is then carried to the east coast of the US through long-distance transport of air pollution plumes [17, 18]. The starkest example of this was the smoke event June 2023 when plumes of air pollution filled the air in New York State [19]. A survey of New York State residents found that 25% of participants reported an illness related to wildfire smoke exposure during the June 2023 event and 87% reported at least one symptom as a result of wildfire smoke exposure. The most commonly reported symptoms included itchy, irritated, watery eyes, sore or irritated throat, and headaches [20]. Furthermore, there has also been an increase in the number of local wildfires and bushfires documented in areas close to major population centers, including Inwood Hill Park in Manhattan, Highbridge Park in the Bronx, Prospect Park in Brooklyn, Sterling Forest in upstate New York, and the Pine Barrens in Long Island.

According to the New York Department of Environmental Conservation, New York State had the highest number of wildland acres burned per fire in decades in 2024, suggesting larger and stronger local fires [21]. However, studies on the health effects of wildfires in the Northeast region of the US are very limited. Studies that exist tend to focus on large metropolitan and urban areas such as New York City [22, 23]. Suburban areas in the Northeast remain largely understudied. Suffolk County, in Long Island, New York is located approximately sixty miles outside of New York City with different demographic and socioeconomic characteristics as compared to the city itself but still accounts for a substantial (7.6%) proportion of the state population [24]. Previous research has shown an increase in the number of and size of wildfires on Long Island over the past three decades, as has the probability of wildfire occurrence [16]. Prior research has also found differences in health outcomes between different areas with varying levels of urbanicity [25]. It is therefore important to study how the health impacts of exposure to wildfire-generated air pollution in specific populations. This will in turn allow for the creation of local and community-specific measures and interventions.

In this study, we have investigated the local effects of a specific wildfire smoke event, the June 2023 Canadian wildfire, on local inpatient hospital admissions and emergency department (ED) visits in a suburban setting in New York State. We further extended our study timeline to look at the effect of short-term exposure to wildfire smoke on local hospital encounters from 2014 through 2023 using a time series analysis. We looked at all-cause encounters as well as cardiovascular and respiratory visits.

## 2. Methods

**Figure 1.**
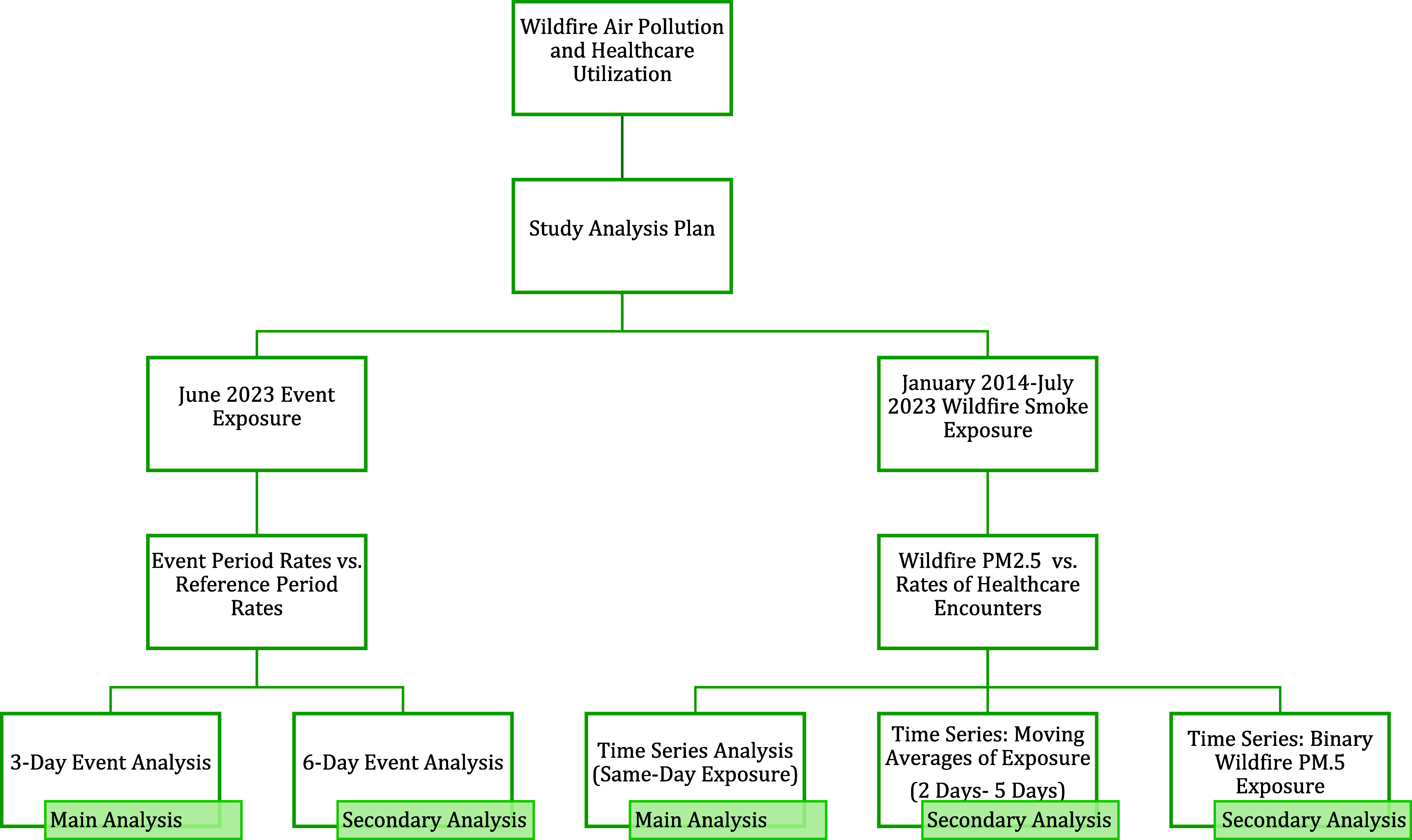
Research design and study analysis plan.

We examined the relationship between exposure to wildfire smoke and local hospital encounters in a suburban setting in New York State. We investigated this relationship in both a specific—and exceptional—wildfire smoke event and also over a multi-year period (figure 1). First, we focused the wildfire smoke event of June 2023 during which air pollution from Canadian wildfires traveled down to New York and blanketed New York City and the surrounding areas. Then, we further extended our study timeline and examined wildfire smoke exposure and local hospital visits between January 2014 and July 2023 using time series analysis. Data on patient characteristics and visits was obtained from the TrinetX platform which derives this information from SBUH electronic health records [26]. This study was approved by the Institutional Review Board at Stony Brook University.

### 2.1. Study population

Our study focused on one of the major health systems in New York: Stony Brook University Hospital (SBUH). SBUH is the main health care provider in Eastern Long Island. It is the only tertiary care center and regional trauma center in the area. With 624 beds, SBUH receives over 100 000 ED visits and over 30 000 inpatient discharges per year [27]. Patients who came to this institution for ED visits or as inpatient hospital admissions between 1 January 2014, and 31 July 2023, were included in the study. Only one admission per day was counted for each individual as it was assumed that admissions on the same day were most likely part of a single medical visit.

### 2.2. Exposure

Our data on wildfire smoke PM_2.5_ came from a publicly available dataset that is widely used in epidemiological research [28, 29]. The model’s developers attribute PM_2.5_ to wildfire-smoke at EPA monitoring stations by combining satellite-identified smoke plumes with ground-based PM_2.5_ observations. They use NOAA’s Hazard Mapping System (HMS) smokeplume polygons, which are daily plume boundaries drawn by analysts from satellite imagery, to determine if a monitor location is impacted by smoke on a specific day. A station–day is identified as smoke-affected if it intersects any HMS plume that day. Given these smoke-day classifications, the researchers then estimated a location-and month-specific non-smoke baseline to represent what PM_2.5_ would have been in the absence of smoke for this monitoring location. Specifically, for each monitor, month, and year, they compute the median PM_2.5_ on non-smoke days within a three-year window (year−1 to year + 1) for that same month. Smoke-attributable PM_2.5_ on a monitor-day is then defined as the difference between observed PM_2.5_ and this non-smoke median on smoke days (and set to zero on non-smoke days). This rolling, month-specific median approach flexibly accounts for local seasonality and gradual trends in background (non-smoke) PM_2.5_ without imposing a parametric time-series model [28, 29]. This smoke PM_2.5_ dataset is created using machine learning methods and data from various remote sensing and ground measurement sources to estimate levels of daily wildfire PM_2.5_ between 2006 and 2023 on a 10 km grid cell. We extracted values from this model at the county level, as provided by the original authors of the model, for Suffolk County, NY. Stony Brook Hospital, as SBUH is located in Suffolk County and serves patients all across the county.

### 2.3. Outcomes

Our outcomes of interest included rates of all-cause hospital encounters which were comprised of inpatient admissions and ED visits. We further specifically looked at cardiovascular disease (CVD), hypertension (HTN), respiratory disease, and asthma. These outcomes were chosen because of their established relevance to the health outcomes associated with air pollution exposure [30]. This data was obtained from the TrinetX platform [26]. The outcomes were defined using the International Classification of Disease (ICD) codes-9 (for visits before 1 October 2015) and ICD codes-10 (for visits on 1 October 2015 and beyond). CVD was defined as ICD-9 codes ‘390–459’ and ICD-10 codes beginning with ‘I’. Hypertension was defined as ICD-9 code ‘401’ and ICD-10 code ‘I10’.

Respiratory disease was defined as ICD-9 codes ‘460–519’ and ICD-10 codes beginning with ‘J’. Asthma was defined as ICD-9 code ‘493’ and ICD-10 code ‘J45’. We defined these codes as outcomes regardless of their position in the list of diagnoses an individual had during a visit.

In order to obtain rates of outcome, we divided counts by population in the county for that year as reported by the US Census Bureau. For 2023, this number was 1523 170 individuals [31, 32].

### 2.4. Covariates

Time-series models are robust to many forms of confounding as ‘time’ is the unit of analysis. Confounders in the case of time series analyses are those that would vary day-to-day as well as seasonal trends [33]. We included a number of time-varying environmental covariates in our model to account for confounding and increase the precision of our effect estimates. Our environmental covariates included other air pollutants such as non-smoke PM_2.5_ and O3 as well as meteorological variables such as average daily temperature, precipitation, and windspeed, downloaded from the Global Historical Climatology Network (GHCN)- which is available from the National Oceanic and Atmospheric Administration (NOAA) [34, 35]. Total PM_2.5_ and O3 were derived from the Environmental Protection Agency’s Air Quality System (EPA AQS) monitors, which were closest to the hospital location [36]. Non-smoke PM_2.5_ was calculated by subtracting smoke PM_2.5_ from the total measured PM_2.5_. Missing covariate data was filled in using linear interpolation.

We further added sine and cosine terms for day of year to account for seasonality in our model. Finally, our model included indicator terms for day of week and holidays.

### 2.5. Statistical analyses

*Wildfire Smoke Event Analysis (June 2023)*

We calculated the standardized incidence rate ratio (SIR) for each of the outcomes listed above by comparing the number of total healthcare encounters, whether in the ED or as an inpatient, during the smoke event (6th June^—^8thJune, 2023) to reference periods defined as comparable days of the week the prior week (30th May^—^1st June 2023) and the week after (13th June^—^15th June 2023). This is comparable to a previous study of this event focused on asthma ED visits in New York City [22]. In this analysis we assume that given the unexpected nature of the event, there are no other factors aside from the wildfire smoke that would account for the differences between the number of visits on event days as compared to reference days. This is a reasonable assumption as the reference periods are the week before and after the event in the same year.

We hypothesized that changes in the rates of the outcomes may also present a few days after the smoke event. As such we also ran the analyses with the event period extended by three days (6th June–11th June 2023) and the reference periods extended (30th May–5th June 2023, and 13th June^—^18th June 2023) by three days each. The choice of three-day moving average window is commonly used in prior research [37].

*Time Series Analysis (January 2014–July 2023)*

In the second portion of the study, we extended the timeline of the exposure-outcome relationship. We conducted a time-series study looking at the relationship between daily exposure to wildfire smoke PM_2.5_ and daily rates of healthcare encounters with the outcomes described above, using the following equation:

\begin{align*} {\mathrm{log(counts}}\,\,{\mathrm{of}}\,\,{\mathrm{outcom}}{{\mathrm{e}}_i}) &amp; = {\beta _0} + {\beta _1}{\mathrm{smoke}}\,\,{\mathrm{P}}{{\mathrm{M}}_{2.5i}} + ns({\mathrm{follow}} - {\mathrm{up}}\,{\mathrm{day}}, df = 3) \nonumber\\ &amp;\quad + {\beta _x}{\mathrm{Covariate}}{{\mathrm{s}}_i} + {\mathrm{offset}}(\log ({\mathrm{populatio}}{{\mathrm{n}}_i})) \end{align*}

where *i* is the day of follow-up and ‘*ns*’ represents a natural cubic spline with three degrees of freedom to represent non-linear time. We specified a quasi-Poisson distribution to allow for overdispersion of the outcome. We ran an additional analysis using a binary smoke PM_2.5_ variable instead of a continuous one which was defined as greater than or equal to the 90th percentile or less than the 90th percentile of smoke PM_2.5_ values. We further looked at moving averages of exposure up to five days to identify critical exposure windows. We also ran sensitivity analyses that checked for an interaction between smoke PM_2.5_ and average temperature and an analysis excluding data from 2020 where there was a noticeable drop in hospital encounters due to the pandemic (figure S1).

All analyses were carried out using R statistical software version 4.4.1 [38]. The SIRs were calculated using the ‘fmsb’ package [39].

## 3. Results

The demographic characteristics of the population with inpatient or emergency visits to Stony Brook Hospital for the Jun 2023 smoke event (and reference periods) can be seen in table 1(A). Men represented a greater proportion of CVD visits and women represented two-thirds of asthma visits. Those who identified as White represented a higher portion of CVD visits while those who identified as Black constituted a higher proportion of respiratory visits as compared to all-cause visits. The demographic characteristics of the population with inpatient or ED visits from January 2014 to July 2023 can be seen in table 1(B). The mean age of patients during this multi-year period was 37 years; slightly greater than half were female; 64% were White; and 7.6% were Black.

**Table 1.** (A) Characteristics of study population-smoke event June 2023 and reference periods. (B) Characteristics of Study Population-Stony Brook Hospital, NY Jan 2014-July 2023.

(A)						

Characteristics		Overall visits	CVD visits	HTN visits	Respiratory visits	Asthma visits
Total events		3906	448	236	277	101

Number of events (smoke event)		1265	180	100	103	40

Number of events (pre-smoke period)		1319	134	65	99	37

Number of events (post-smoke period)		1322	134	71	75	24

Number of individuals		3669	439	233	272	98

Age at baseline (years)^a^		42.07 (24.78)	65.10 (16.78)	64.36 (14.85)	38.67 (28.04)	33.14 (23.13)

Sex^b^	Female	2027 (55.2%)	205 (46.7%)	120 (51.5%)	146 (53.7%)	65 (66.3%)
	Male	1640 (44.7%)	234 (53.3%)	113 (48.5%)	126 (46.3%)	33 (33.7%)

Race^b^	White	2267 (61.8%)	337 (76.8%)	170 (73.0%)	161 (59.2%)	54 (55.1%)
	Black	319 (8.7%)	30 (6.8%)	22 (9.4%)	36 (13.2%)	15 (15.3%)
	Unknown/ Other	1083 (29.5%)	72 (16.4%)	41 (17.6%)	75 (27.6%)	29 (29.6%)

(B)						

Characteristics		Overall visits	CVD visits	HTN visits	Respiratory visits	Asthma visits

Number of events		1285 631	171 971	86 481	120 759	40 097

Number of individuals		517 242	95 664	54 750	77 894	23 237

Age at baseline (years)^a^		37.55 (23.50)	59.56 (17.23)	60.85 (14.58)	34.70 (26.94)	28.93 (21.42)

Sex^b^	Female	268 488 (51.9%)	45 149 (47.2%)	26 996 (49.3%)	39 661 (50.9%)	13 198 (56.8%)
	Male	248 331 (48.0%)	50 473 (52.8%)	27 736 (50.7%)	38 170 (49.0%)	9998 (43.0%)

Race^b^	White	332 597 (64.3%)	74 702 (78.1%)	41 414 (75.6%)	51 558 (66.2%)	148 84 (64.1%)
	Black	39 272 (7.6%)	6691 (7.0%)	4499 (8.2%)	7454 (9.6%)	3075 (13.2%)
	Other/ Unknown	145 373 (28.1%)	14 271 (14.9%)	8837 (16.1%)	18 882 (24.2%)	5278 (22.7%)

a Mean (standard deviation).

b Counts (%).

**Figure 2.**
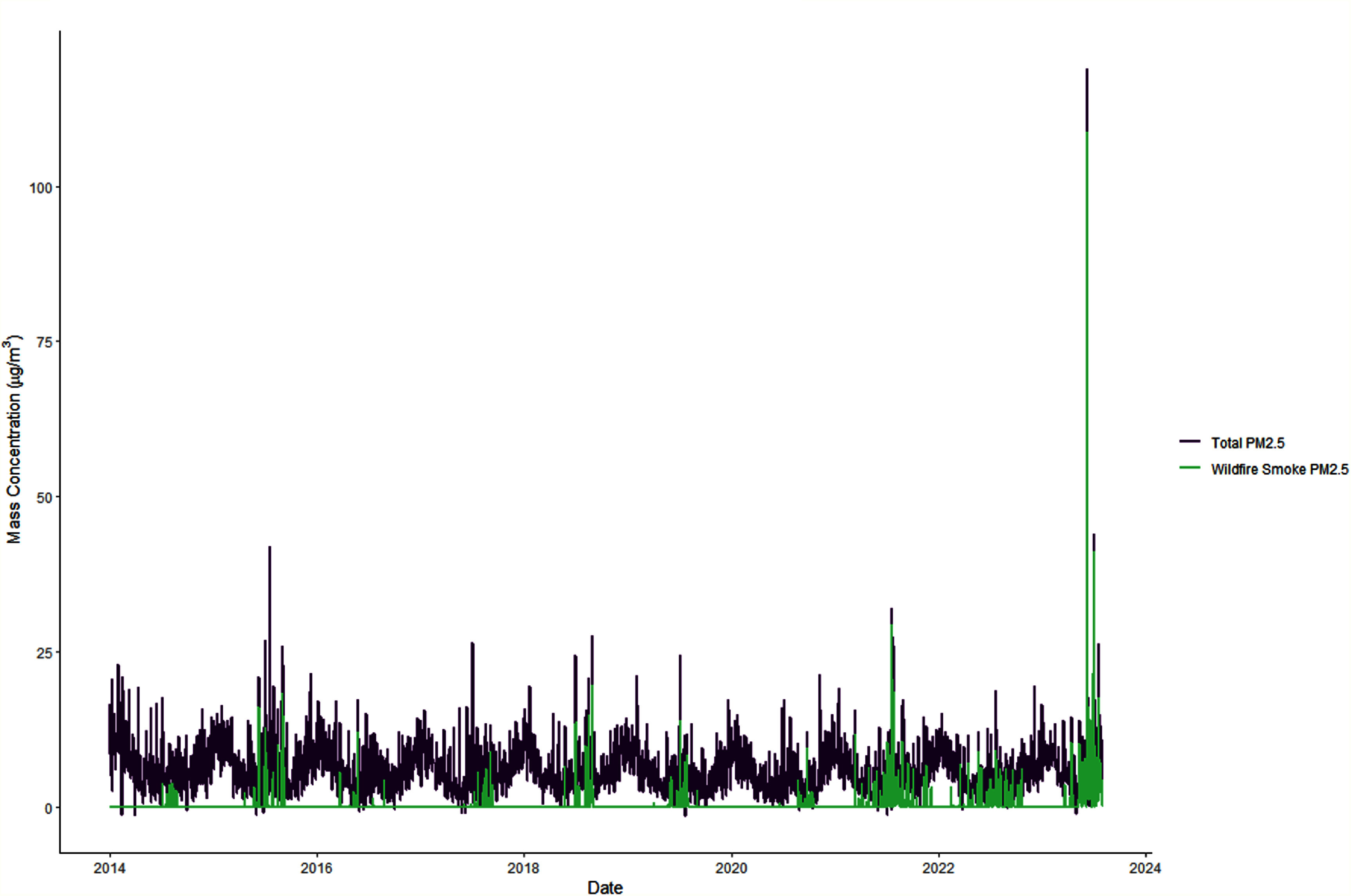
Total PM_2.5_ and wildfire-associated PM_2.5_ in Suffolk County, New York (January 2014–June 2023).

The distribution of exposure over the study period can be seen in figure 2. There is a noticeable spike in overall and wildfire-associated PM_2.5_ levels that occurred in June 2023 during the smoke episode. The distribution for our other environmental variables can be seen in table S1. Consistent with the seasonal nature of weather in Suffolk County, NY, environmental conditions vary throughout the year. Median air pollution levels tend to be lower than current regulatory standards.

The results of the first portion of our analysis can be seen in tables 2(A) and 2(B). We found increased rates of all-cause hospital encounters and ED visits for CVD and hypertension during the wildfire smoke event, as well as inpatient admissions with respiratory disease (table 2(A)). When we extended the event and reference periods by three days each, the results largely remained the same except for total respiratory encounters which were now significantly increased (rate ratio: 1.22 (95% CI: 1.03–1.45)). In both cases, all-cause ED visit rates were lower during the wildfire smoke event than during the reference periods (table 2(B)).

**Table 2.** (A) Main results-standardized incidence rate ratios during smoke event 2023. (B) Results for extended event time frame-standardized incidence rate ratios during smoke event 2023.

(A)			

Outcome	All visits^a,b^	Emergency visits^a,b^	Inpatient admissions^a,b^
All	0.96 (0.90–1.02)	**0.92 (0.86–0.99)**	1.01 (0.87–1.16)
CVD	**1.34 (1.11–1.62)**	**1.52 (1.20–1.93)**	1.10 (0.81–1.50)
HTN	**1.47 (1.14–1.90)**	**1.47 (1.11–1.96)**	1.46 (0.81–2.64)
Respiratory	1.18 (0.93–1.51)	1.04 (0.77–1.40)	**1.60 (1.03–2.48)**
Asthma	1.31 (0.88–1.95)	1.19 (0.77–1.84)	2.29 (0.83–6.30)

(B)			

Outcome	All visits^a,b^	Emergency visits^a,b^	Inpatient admissions^a,b^

All	0.98 (0.94–1.03)	**0.94 (0.89–0.99)**	1.06 (0.95–1.18)
CVD	**1.29 (1.12–1.49)**	**1.51 (1.26–1.80)**	1.00 (0.79–1.27)
HTN	**1.52 (1.25–1.85)**	**1.56 (1.26–1.94)**	1.36 (0.86–2.17)
Respiratory	**1.22 (1.03–1.45)**	1.12 (0.91–1.38)	**1.50 (1.09–2.06)**
Asthma	1.26 (0.93–1.72)	1.23 (0.89–1.71)	1.38 (0.59–3.24)

a Standardized incidence rate ratios (95% Confidence intervals).

b Bold font indicates significant results where the 95% confidence interval does not cross 1.00.

**Figure 3.**
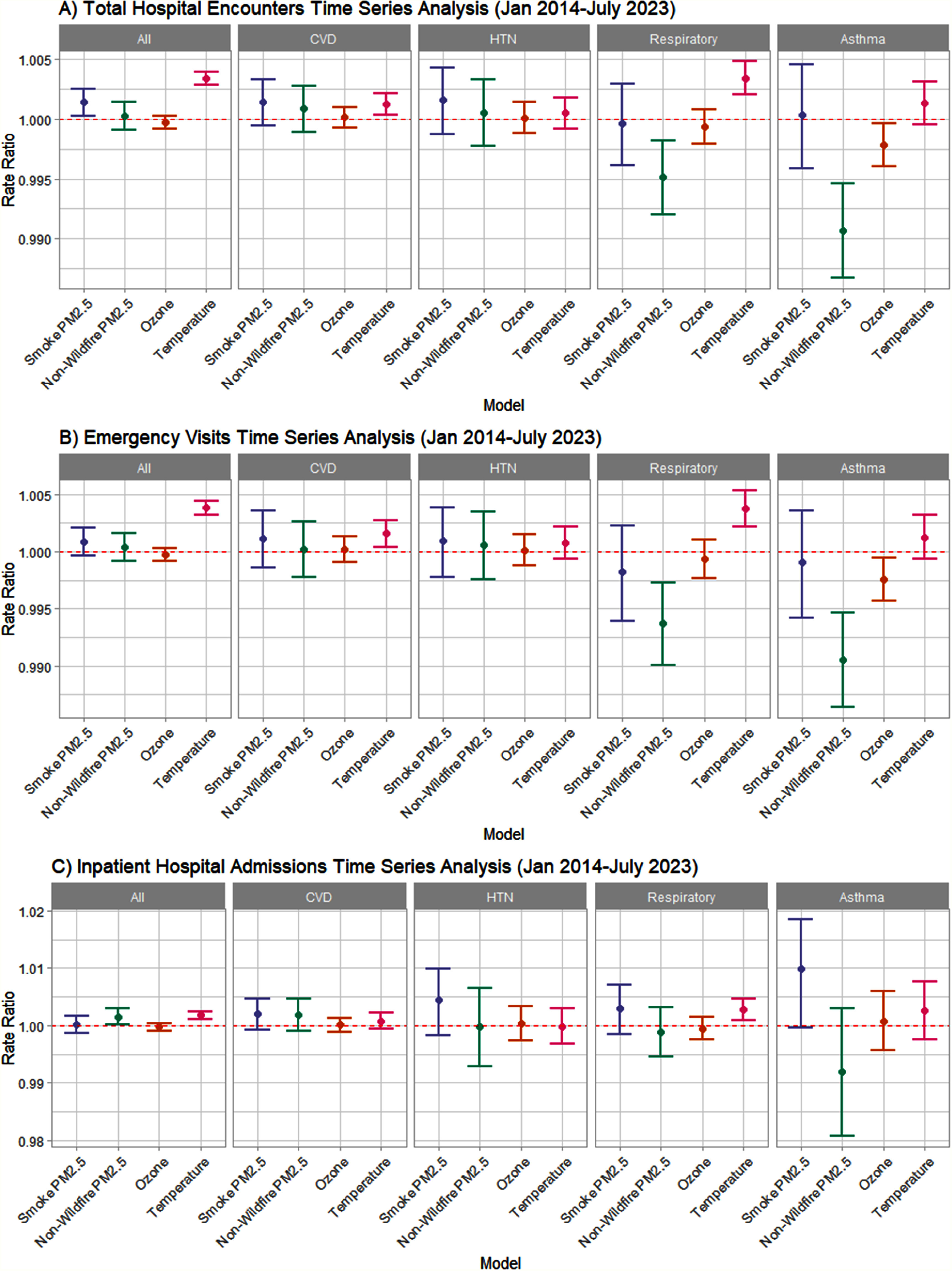
Main results-time-series analyses (January 2014–June 2023) (A) total hospital encounters time series analysis (January 2014–July 2023) (B) emergency visits time series analysis (January 2014–July 2023) (C) inpatient hospital admissions time series analysis (January 2014–July 2023).

In the second multi-year portion of our study, we conducted a time series analysis of daily ED visits and inpatient admissions and estimated levels of wildfire-generated PM_2.5_ between 2014 and 2023. The results can be seen in figure 3. We found slightly elevated rates of all-cause hospital visits with exposure to smoke PM_2.5_.

For total hospital encounters and ED visits alone, non-smoke PM_2.5_ exposure was associated with a slightly protective effect for respiratory and asthma visits. For inpatient admissions alone, non-smoke PM_2.5_ exposure was associated with increased rates of all-cause admissions We found slightly protective effects for ozone for total hospital encounters and ED visits with asthma. For inpatient admissions alone, ozone was not associated with any of the outcomes measured (figure 3).

Higher temperature was associated with higher rates of all-cause visits, CVD visits, and respiratory visits when looking at total healthcare encounters and ED visits. For inpatient admissions alone, higher temperatures were associated with higher rates of all-cause admissions and respiratory admissions (figure 3).

We ran analyses using a binary measure of smoke PM_2.5_ as our exposure of interest and found that rates of total encounters, ED visits, inpatient visits for all-causes and inpatient CVD admissions increased with higher smoke PM_2.5_ levels (table 3).

**Table 3.** Results using binary smoke PM_2.5_ definition.

	All visits^a,b^	Emergency visits^a,b^	Inpatient admissions^a,b^
All Admissions	**1.03 (1.02–1.04)**	**1.03 (1.01–1.04)**	**1.02 (1.00–1.03)**
CVD	1.01 (0.99–1.03)	0.99 (0.96–1.01)	**1.04 (1.01–1.07)**
HTN	0.99 (0.96–1.02)	0.98 (0.95–1.01)	1.03 (0.96–1.11)
Respiratory Disease	0.98 (0.95–1.02)	0.98 (0.94–1.03)	0.99 (0.94–1.04)
Asthma	0.99 (0.94–1.04)	0.99 (0.95–1.04)	0.96 (0.84–1.09)

a Rate ratios (95% Confidence intervals).

b Bold font indicates significant results where the 95% confidence interval does not cross 1.00.

The results of the analysis of total hospital encounters using moving averages of exposures can be seen in figure S2. Smoke PM_2.5_ was associated with increased rates of all-cause healthcare encounters across all moving averages with higher rates generally seen over the longer moving averages. Non- smoke PM_2.5_ was associated with lower rates of respiratory and asthma visits across moving averages. Temperature was associated with increased rates of all-cause healthcare encounters and respiratory diseases across all studied exposure windows. When looking at ED visits alone across moving averages of exposure, similar trends were observed for non-smoke PM_2.5_ and temperature (figure S3). For inpatient visits alone, we found increased rates of all-cause visits for non-smoke PM_2.5_ and temperature across exposure time windows. We also found increased rates of inpatient admission for respiratory disease with higher temperature exposure (figure S4).

In our sensitivity analysis which excluded data from the year 2020, as it led to fluctuations in hospital visits, we found largely similar results to our main analysis with the exception of ozone, which was found to be slightly protective of all-cause visits and respiratory visits (figure S5). Finally, we looked at the interaction between smoke PM_2.5_ and temperature. We found significant interactions for all-cause visits and cardiovascular visits. In those models, smoke PM_2.5_ was no longer significantly associated with increased rates of all-cause visits, but did significantly increase total CVD visits (figure S6).

## 4. Discussion

In this study, we examined changes in healthcare encounters, ED visits, and inpatient admissions, during the wildfire smoke event of June 2023 in a suburban setting in New York State. We further extended our study period back to 2014 to study the association of wildfire-generated PM_2.5_ on local health outcomes using a time series analysis. We found that overall encounters and ED visits with CVD and HTN increased during the smoke episode of June 2023. Inpatient admissions with respiratory diseases increased as well. These trends persisted when extending the event and reference periods a further three days. There is also a serious financial burden associated with these kinds of wildfire events. Using data we previously obtained from the Healthcare Utilization Project (HCUP), we found that the average amount charged by hospitals in the case of an ED visit with a cardiovascular primary diagnostic code in 2018 in the state of NY was over $6200 [40]. After adjustment for inflation, this translates into $7880 in 2026 [41]. We estimated an extra 40 additional CVD ED visits at our institution during the wildfire period as compared to reference periods. This amounts to an extra cost of over $315 000 at a single hospital ED over a period of three days in response to this wildfire event. Multiplied across outcomes and geographic areas, these events pose a significant cost burden to the health care system.

In our time series analyses, we also found increased rates of all-cause hospital visits associated with exposure to higher levels of smoke PM_2.5_. When using a binary definition of smoke PM_2.5_, we also saw increased rates of inpatient CVD admission with higher exposures.

Non-smoke PM_2.5_ increased the rate of all-cause inpatient visits and had a protective effect for overall and ED respiratory and asthma visits. Ozone generally had a slightly protective effect for overall and ED asthma visits. Higher temperature was associated with increased rates of all-cause, cardiovascular, and respiratory total encounters and ED visits. Our analysis using moving averages of exposures showed that the adverse effects of smoke PM_2.5_ and temperature lasted across several days.

Some of our results are comparable to previous results looking at the effects of wildfire-generated air pollution and health outcomes. The most direct comparison to our work is a study looking at the effects of June 2023 smoke event on ED visits for asthma in NYC using similar methods. This study found an increase in the rate of admissions for asthma during this event (Rate Ratio: 1.44, 95% CI: 1.31–1.58) [22]. We also found an increase in the rate of asthma ED visits (Rate Ratio: 1.19 (95% CI: 0.77–1.84)), although our result was not statistically significant. In terms of absolute value, the rate ratio for emergency asthma visits was lower than that of NYC. This could be due to the protective effects of higher socioeconomic status in Suffolk County. Another study of asthma ED visits in NYC found a 2% increase in the rate of visits per 10 *µ*g m^−^3 increase in the level of wildfire smoke PM_2.5_ during this specific event [23]. A study looking at EMS calls in NYC during the wildfire event found an increased number of calls for respiratory problems and treatments for chest pain but not an increase in calls for cardiovascular conditions [42], which is contrary to what we found in our research as we saw increases in ED visits with cardiovascular conditions. This study did however report an increase in EKG-12 lead use by EMS, which may indicate some misclassification in the designation of calls [42]. A study in Maryland also found increased cardiopulmonary encounters during the 2023 smoke event [43]. A study looking at the 2023 Canadian wildfires found that approximately 5400 (95% CI: 3400–7400) excess acute deaths in North America could be attributed to the wildfires [44]. In Canada itself, where these wildfire originated, researchers also found increased asthma-related ED visits [45]. We also found an increase in the rate of inpatient respiratory admissions during the smoke event in Suffolk County, NY. These studies and ours highlight the wide-ranging effects of a single wildfire event. As events similar to this one are expected to increase, there must be greater urgency of action on mitigation and adaptation strategies that best address the needs of local communities.

Outside of the specific June 2023 event, previous studies have also found adverse health outcomes related to wildfire smoke exposure. Studies on the acute health effects of wildfire-generated air pollution have mostly come from the western region of the US. A study of ED visits in California between 2006 and 2017 found increases in visits for respiratory conditions but not cardiovascular disorders in response to wildfire smoke exposure [46]. A time-series study from Colorado from 2010 to 2015 found elevated odds of cardiac arrest deaths, respiratory and asthma hospitalizations with exposure to wildfire smoke [37]. A study of cardiorespiratory hospital visits in California from the 2004 to 2009 wildfire seasons revealed higher rates of encounters for respiratory diseases and asthma. The researchers did not find a significant increase in cardiovascular visits in the overall population as we did in ours. They did, however, find increased rates of cardiovascular visits in vulnerable subgroups [1]. A study of the association between wildfire air pollution and CVD in California in 2018 found an increased rate of both CVD events (Rate Ratio: 1.231 (95% CI, 1.039–1.458)) and CVD deaths (Rate Ratio: 1.358 (95% CI, 1.128–1.635)) [47]. In a time-stratified case-crossover study of ED visits from five western states between 2007 and 2018, researchers found increased odds of visits for asthma associated with increased wildfire PM_2.5_ exposure [48]. These and other studies have found extensive respiratory effects in response to exposure to wildfire smoke but results concerning CVDs have been more inconsistent [49, 50]. Meanwhile our study found mixed results depending on whether we were looking at ED visits or inpatient admissions. This could be due to several reasons. Firstly, there is a difference in the composition/properties of wildfire air pollution generated locally in the West as compared to that which is the result of long-range transport in the East. Secondly, the age distribution of our CVD patients and respiratory admissions vary widely. It is possible that the older population with CVDs are more likely to experience exacerbations in response to air pollution events than younger patients with respiratory diseases. Most of the smoke days in Suffolk County occurred after 2019 which is in contrast to the West Coast, which has dealt with wildfires for many years. Therefore, their existing adaptation strategies may influence the adverse health outcomes encountered. There may also be additional residual or unmeasured confounding. Finally, our study might be underpowered to detect some of the health effects as it only includes data from a single location. These discrepancies also highlight the need for research in specific populations as vulnerabilities may vary.

Our study had several limitations. We only had data on a single hospital site. As such, the results may not be generalizable to areas that are not similar to Suffolk County, New York. We assumed that all patients visiting the SBUH ED were residents of Suffolk County, which cannot be verified based on our data. However, given the size of the county, anyone not living in Suffolk County is probably from a neighboring county which would have similar exposure levels. Furthermore, our small sample size limited our ability to conduct any subgroup analyses to identify specific vulnerable populations in our community and data on specific characteristics were missing for a considerable portion of the population. Our ascertainment of outcomes was through ICD codes in a manner which did not allow us to identify whether it was the primary reason for the encounter or an underlying condition. Moreover, using modeled and aggregated pollution data may result in exposure measurement error; we would expect this error to be non-differential and biased towards the null [51]. Our study time period overlapped with the pandemic which could have affected hospital utilization. In order to account for this, we ran an additional analysis excluding the year 2020 during which there was a notable drop in hospital visits. Finally, as this is an observational study we could not establish a causal relationship between the exposure and the outcome.

This study also had several strengths. The study setting is within the Northeast region of the US which is understudied as wildfires have only recently become a significant source of air pollution in this region. It focused on the local effects of wildfire smoke PM_2.5_ in a suburban area, which have been understudied previously, but which can identify specific concerns in areas which have similar characteristics. We looked at changes in hospital encounters for both a specific high air pollution event caused by wildfire smoke and we looked at trends over time. The time-series design would account for confounding by non-varying or slowly varying variables. For those that do vary day-to-day we accounted for changes in meteorological variables, day of week, holidays, and seasonality. We examined multiple health outcomes and types of healthcare encounters to better understand the exposure-outcome relationship in this community.

## 5. Conclusion

Our results showed that smoke PM_2.5_ during the June 2023 event increased the rate of hospital encounters with CVDs. Our time series analysis showed that exposure to smoke PM_2.5_ was associated with increased rates of hospital encounters. As exposure to wildfire smoke increases in the Northeast region of the US, it is imperative to better understand the health impacts of this exposure on local communities.

## Acknowledgements

This work is supported by the National Institute for Environmental Health Sciences.(NIEHS) Grant R01ES036566. This work is also supported by National Aeronautics and Space Administration (NASA) Grants (80NSSC23K0028) and (80NSSC21K0507).

## Data Availability

The data cannot be made publicly available upon publication because they are owned by a third party and the terms of use prevent public distribution. The data that support the findings of this study are available upon reasonable request from the authors. Supplementary data available at: https://doi.org/10.1088/2515-7620/ae69a4/data1.
